# Calculation for tensile strength and fracture toughness of granite with three kinds of grain sizes using three-point-bending test

**DOI:** 10.1371/journal.pone.0180880

**Published:** 2018-03-29

**Authors:** Miao Yu, Chenhui Wei, Leilei Niu, Shaohua Li, Yongjun Yu

**Affiliations:** School of Resources and Civil Engineering, Northeastern University, Shenyang, China; Beihang University, CHINA

## Abstract

Tensile strength and fracture toughness, important parameters of the rock for engineering applications are difficult to measure. Thus this paper selected three kinds of granite samples (grain sizes = 1.01mm, 2.12mm and 3mm), used the combined experiments of physical and numerical simulation (RFPA-DIP version) to conduct three-point-bending (3-p-b) tests with different notches and introduced the acoustic emission monitor system to analyze the fracture mechanism around the notch tips. To study the effects of grain size on the tensile strength and toughness of rock samples, a modified fracture model was established linking fictitious crack to the grain size so that the microstructure of the specimens and fictitious crack growth can be considered together. The fractal method was introduced to represent microstructure of three kinds of granites and used to determine the length of fictitious crack. It is a simple and novel method to calculate the tensile strength and fracture toughness directly. Finally, the theoretical model was verified by the comparison to the numerical experiments by calculating the nominal strength *σ*_*n*_ and maximum loads *P*_*max*_.

## Introduction

It is well recognized that rock as a heterogeneous brittle material shows a much higher compressive strength than tensile strength (more or less 10 times). Therefore it is important to understand local tensile stress of the rock samples under different mechanical loadings [1–5]. Because of the existing of the microcracks the propagation and coalescence of these defects can easily make the rock samples failure. Therefore, the researches on fracture toughness are also the key problems to the rock mechanical properties and failure modes as important as basic mechanical behaviors [6–9]. Test procedures were suggested by ISRM [10] for rock fracture toughness, where chevron bend (CB) [11], short rod (SR) [12] and cracked chevron-notched Brazilian disk (CCNBD) [13] were adopted as standard specimens. But many scholars [14, 15] thought that neither tensile strength nor fracture toughness can be obtained easily through the physical experiment, and therefore indirect experimental methods such as Brazilian disc and notched three-point-bending samples have been taken into consideration and semi-empirical relations have been built to measure the tensile strength and fracture toughness. Tutluoglu et.al [16] used the three methods (CCNBD, SCB and SNDB) to calculate the fracture toughness of granite and it can be concluded that the growth of fracture process zone is the major factor affecting the experimental results. Dai et al. [17] conducted the numerical simulation to measure the mode I fracture toughness of rocks according to four methods (ISRM suggested). By comparison of the above methods, it can be found that the fracture of the semi-circular bend (SCB) specimen agrees with the measuring principle. Wei et al. [18] analyzed the fracture mechanism of the SCB specimens by both acoustic emission (AE) monitoring and numerical modeling and discussed the relationship between effective crack lengths and fracture toughness. Nasseri et al. [19] used four relatively fine grained and homogeneous granitic rocks to investigate the relationship between their microstructural properties and fracture toughness.

**Fig 1:**
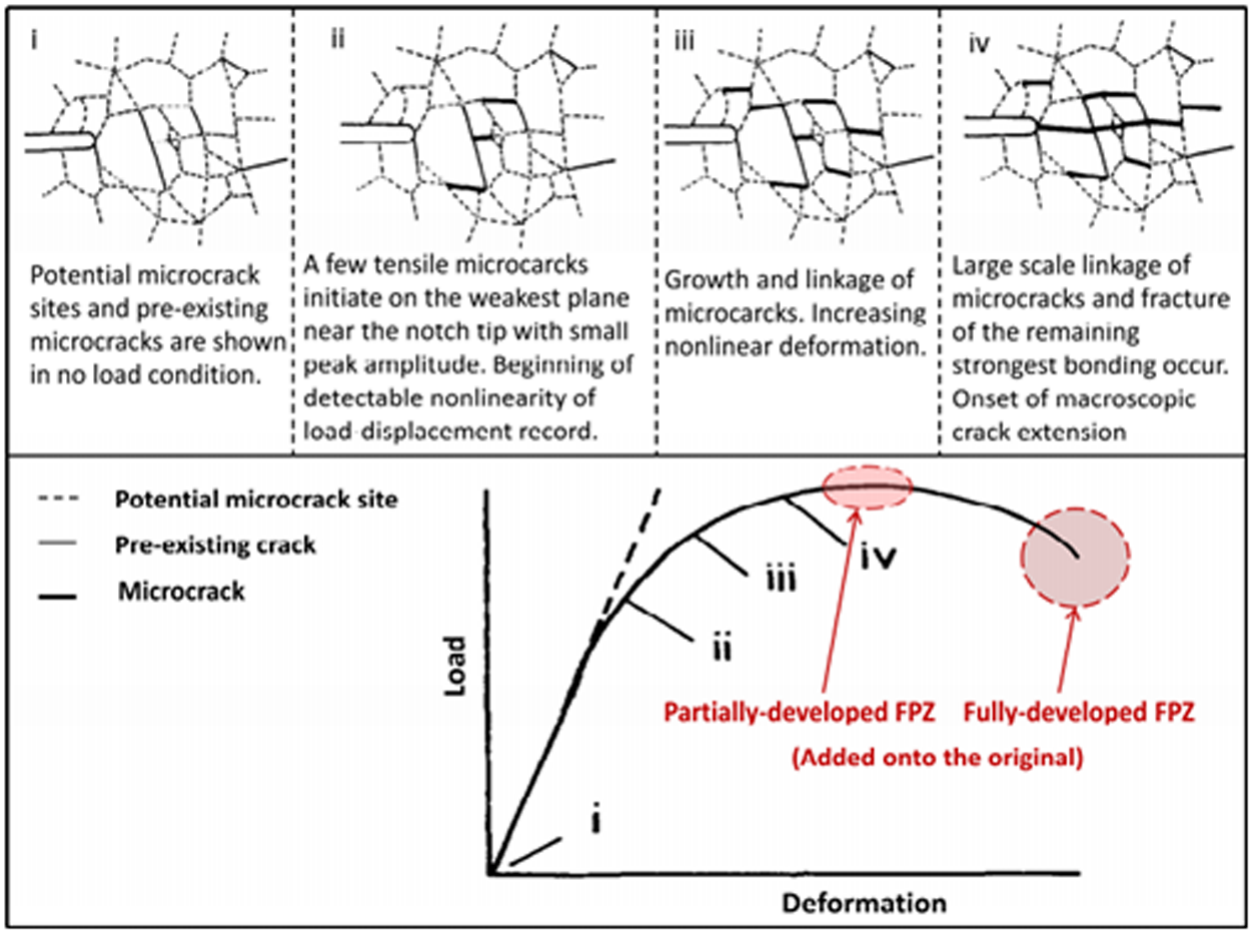
Four fracture phases and load-displacement curve of granite under 3-p-b tests.

Prior to the peak stress under quasi-static loads, the tip of notched crack can appear an inelastic zone, which is defined the fracture process zone (FPZ). It is a hot research field that many professors [20–26] pay attention to by using numerical simulation, theoretical models and physical experiments since Hillerborg [27] proposed the fictitious crack model (or cohesive crack model) to describe the FPZ. During the fracture process the cohesive stresses are used to explain the crack-bridging mechanism due to zig-zag cracking and frictional sliding and pull-out of grains [28]. As shown in Fig 1, fracture process of granite samples can be divided into four phases: (i) potential micro-cracks distribution; (ii) the notch tip of cracks appears damage zones caused by tensile stress which initiates along the load direction regularly; (iii) growth and coalescence between micro-defects and non-linear deformation increases; (iv) occurrence and onset of macro-cracks [29]. It is concluded that FPZ is formed in phase ii where large number of microcracks aggregation and interaction in a local region.

In recent years, the acoustic emission technology is introduced to monitor the damage appearance of rock specimen in order to locate the micro-cracks initiation and growth direction. It has also been applied to the study on the brittle materials under quasi-static loadings [30–32], but few achievements can be found in the field of fracture process.

This paper has taken above questions into consideration, picked the three kinds of granites processed into three-point-bending (3-p-b) samples with length = 280mm, height = 70mm and thickness = 25mm. Moreover, the samples are made different notched lengths in the middle of the beam length raging from short (compared with grain size) to deep (compared with beam height). A modified fracture model is introduced in this study to describe the formation of damage zone and FPZ at the notch tip [33,34], which can be used to calculate the tensile strength and fracture toughness from 3-p-b test. The concept of fractal [35] is used to quantify the meso-scale distribution of the granite and built the relationship between fractal values and fictitious crack growth. Finally, the numerical software of RFPA-DIP version can be used to simulate the fracture process in order to validate the theoretical model, and it can also offer an effective method to understand the failure mechanism of granite combined with acoustic emission results.

## Sample preparation and 3-p-b Test

### Different notched lengths of granite with three kinds of grain sizes

**Fig 2:**
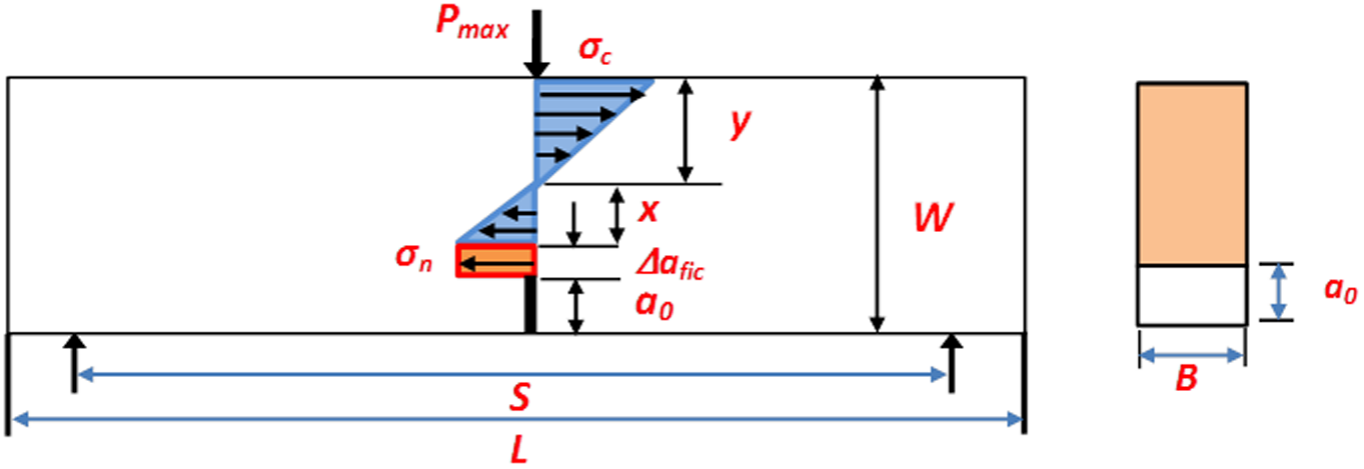
(a) The specimen size of granite with different notched lengths and (b) stress distribution in the crack tips under 3-p-b test.

The samples of granite are picked from Anshan, located in the northeast of China, whose average grain size is 1.01, 2.12 and 3 mm. The granite blocks are processed to cuboid specimen with length = 280mm, height = 70mm and thickness = 25mm as illustrated in Fig 2, which is represented by *L*, *W* and *B* respectively. In the figure, the S is the span of 3-p-b test; *a*_*0*_ represents the notched length prefabricated in the middle of the bottom boundary with length = 0, 1, 5, 10, 15, 20, 25, 30, 35, 40 and 45 mm; *P* is the applied load. The number of samples for each notched length can be prepared to 3-p-b tests listed in Table 1 in the study, which are grouped by three kinds of grain sizes equally.

**Table 1:** Different notched lengths for 3-p-b test.

Notch length *a*_0_(mm)	*α = a*_*0*_*/W*	Samples tested
0	0.000	9
1	0.014	9
5	0.0714	9
10	0.143	9
15	0.213	9
20	0.286	9
25	0.357	9
30	0.429	6
35	0.50	6
40	0.571	6
45	0.643	6

Fig 2(b) is the stress distribution model around the notch tip under 3-p-b test, in which *S* and *W* represents the span of the 3-p-b and height of specimen; *P*_max_ represents the peak load; *a*_0_ represents the notch length; Δ*a*_*fic*_ can be used to describe the fictitious crack growth; *σ*_*n*_ and *σ*_*c*_ are nominal strength and compressive stress of granite samples.

### Experimental apparatus and 3-p-b test results

**Fig 3:**
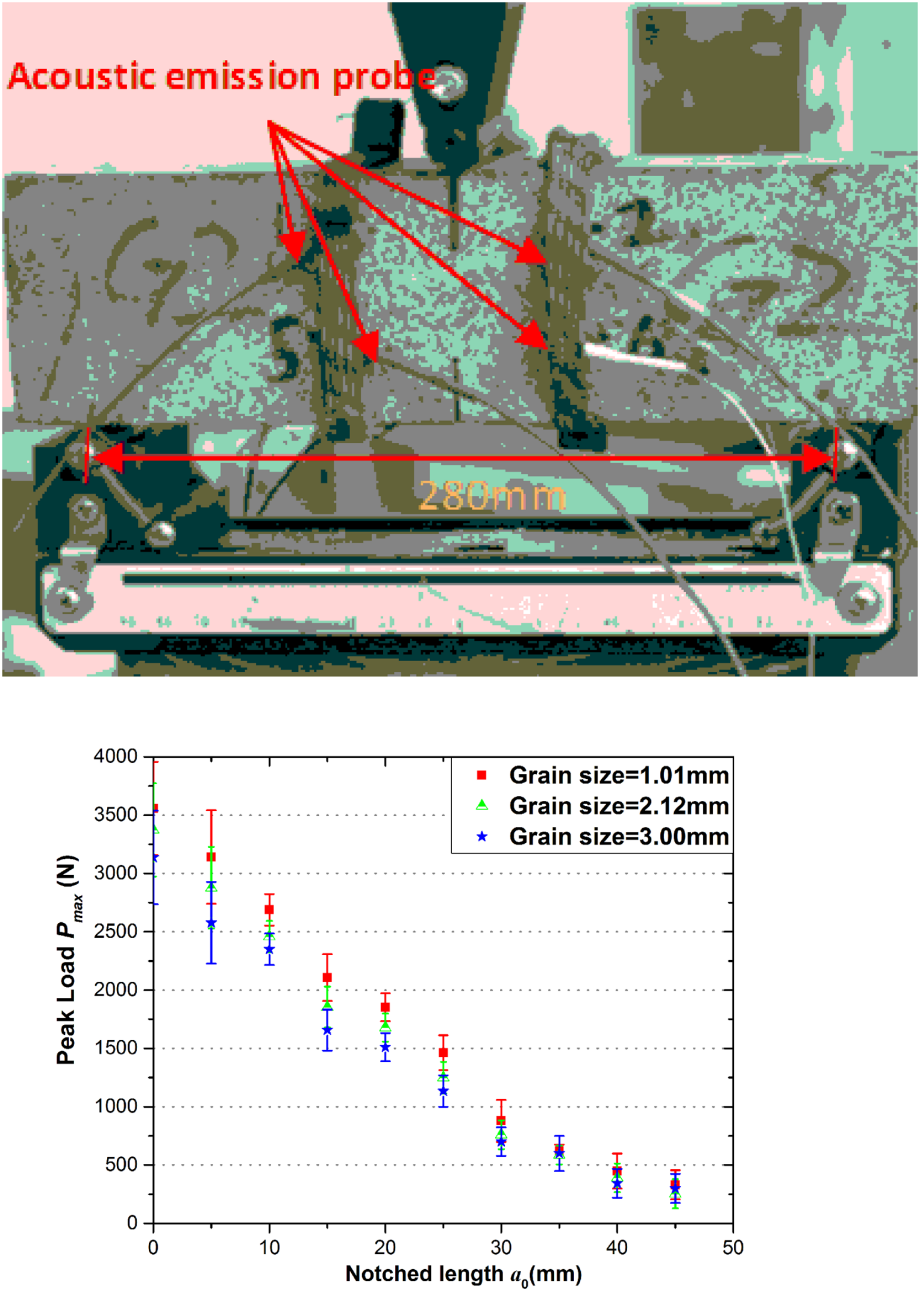
(a) Test loading system of granite under 3-p-b test and (b) Peak loads of three kinds of grain sizes under 3-p-b test.

In this paper, 3-p-b experiments of different grain sizes are conducted by microcomputer controlled electrohydraulic servo-testing system (model TAW-2000KN) produced by Jinli Testing Technology Corporation illustrated in Fig 3(a). Eight probes are split to fix up on either side of the front and back respectively in order to monitor the signal acquisition from the damage formation, micro-crack initiation to the failure of the specimen at the notch tip during the fracture process. Fig 3(b) shows the relationship between the notch length *a*_0_ and peak load *P*_max_ of three kinds of selected granites under 3-p-b tests and the width of the notch is 1-mm-deep through the thickness.

**Fig 4:**
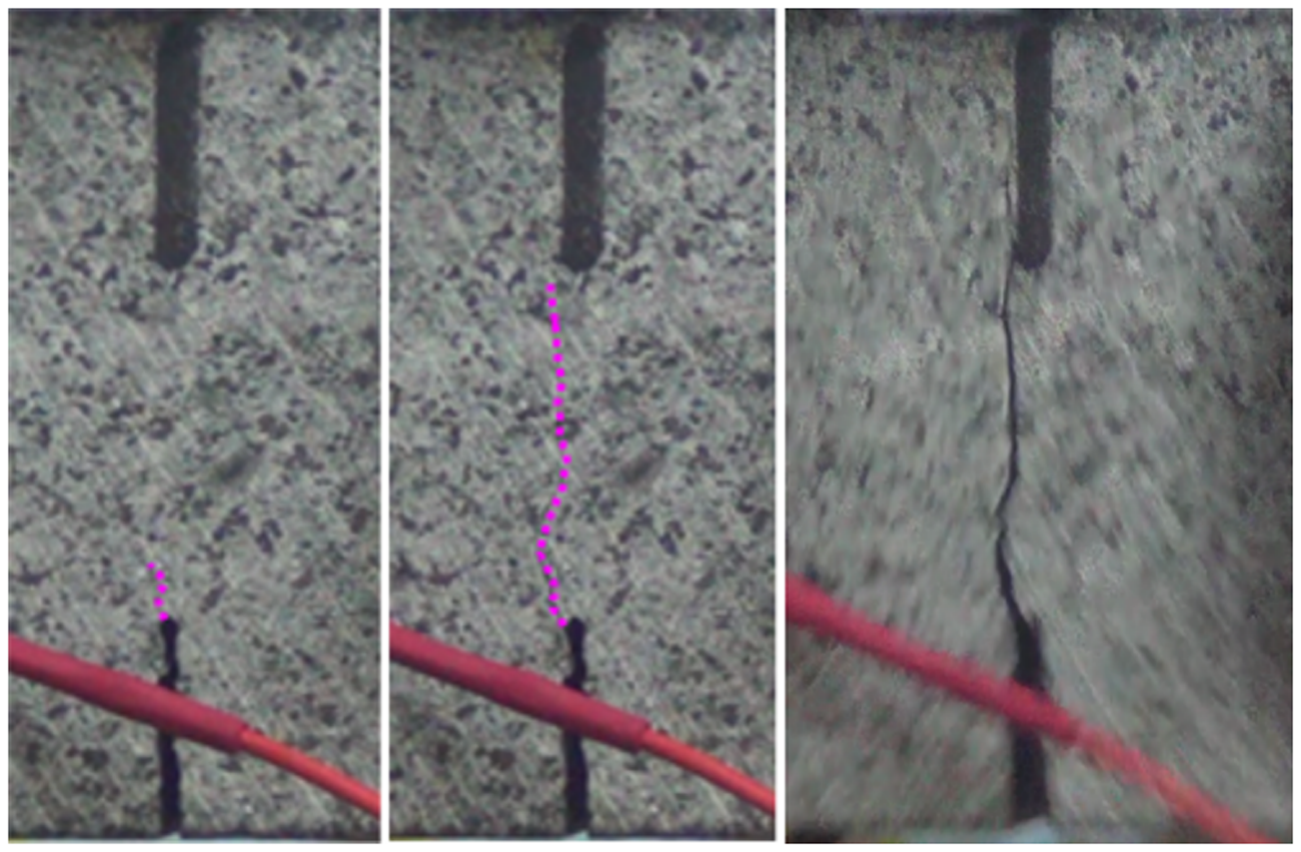
Different fracture phases of granite under 3-p-b test. (a) Formation of fictitious crack (b) Instable growth of micro-cracks (c) Macro-crack growth and coalescence.

Fig 4 represents the following phases of failure process of granite samples under 3-p-b test. It can be seen from the following three figures, the crack propagation paths of the samples are all broken line, which are extended upward along the mineral grain boundaries. In the figure, it can be recognized that the three major phases of the rock fracture process: (1) formation and growth of fictitious crack; (2) instable growth of micro-cracks; (3) macro-cracks growth and coalescence [34].

### Acoustic emission monitor system

**Fig 5:**
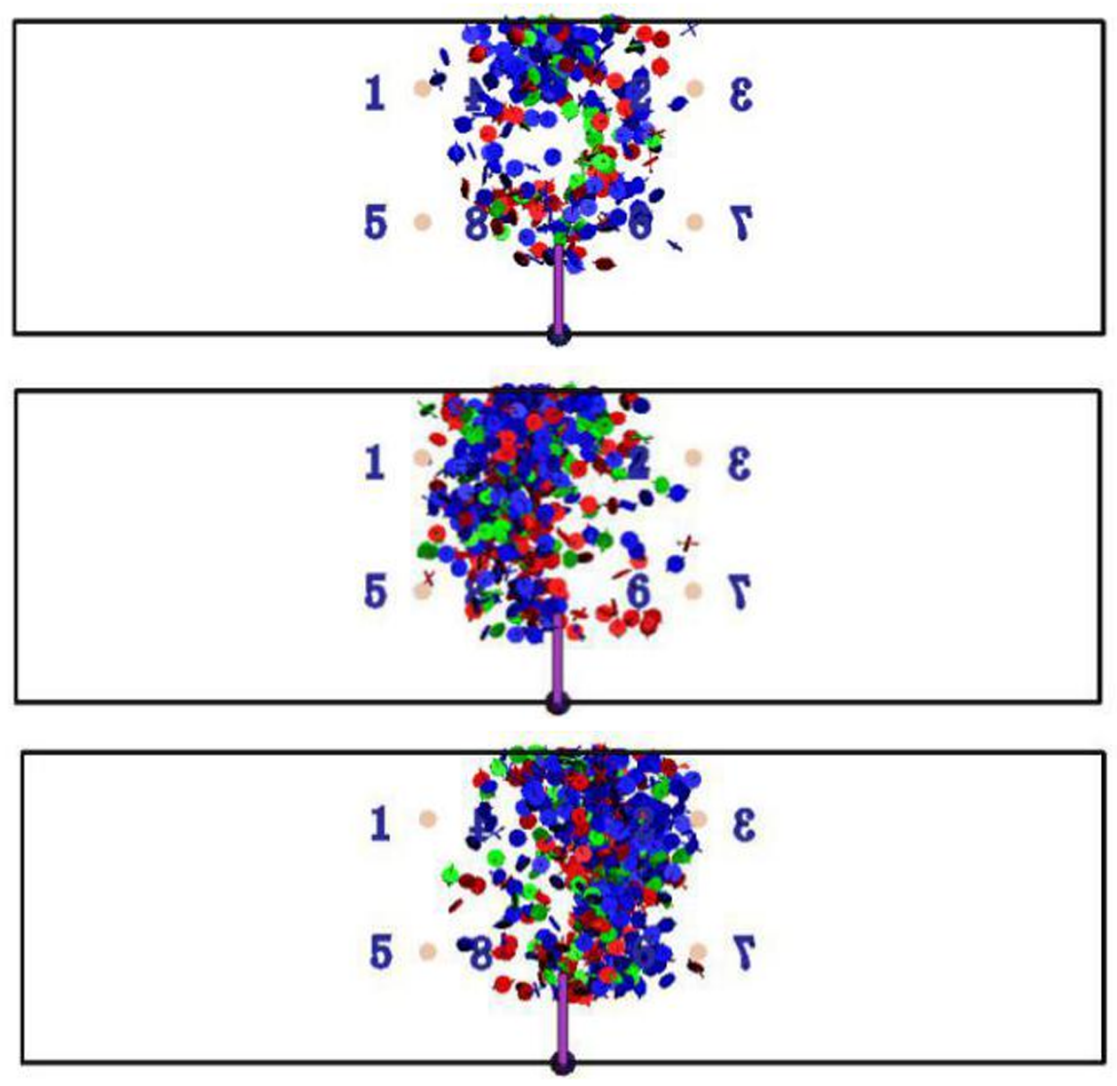
Granite acoustic emission of 3-p-b test with different grain sizes. (a) Fine grain (b) Medium grain (c) Coarse grain.

The moment tensor inversion theory is applied to analyze the meso-failure mechanism of granites with different grain sizes under 3-p-b tests in this paper. The spatial distribution and occurrence probability of shear failure, tensile failure and mixed failure can be obtained by the means of moment tensor analysis methods. Fig 5 represents failure signal of samples with three kinds of grain sizes by the statistical analysis of the moment tensor.

**Fig 6:**
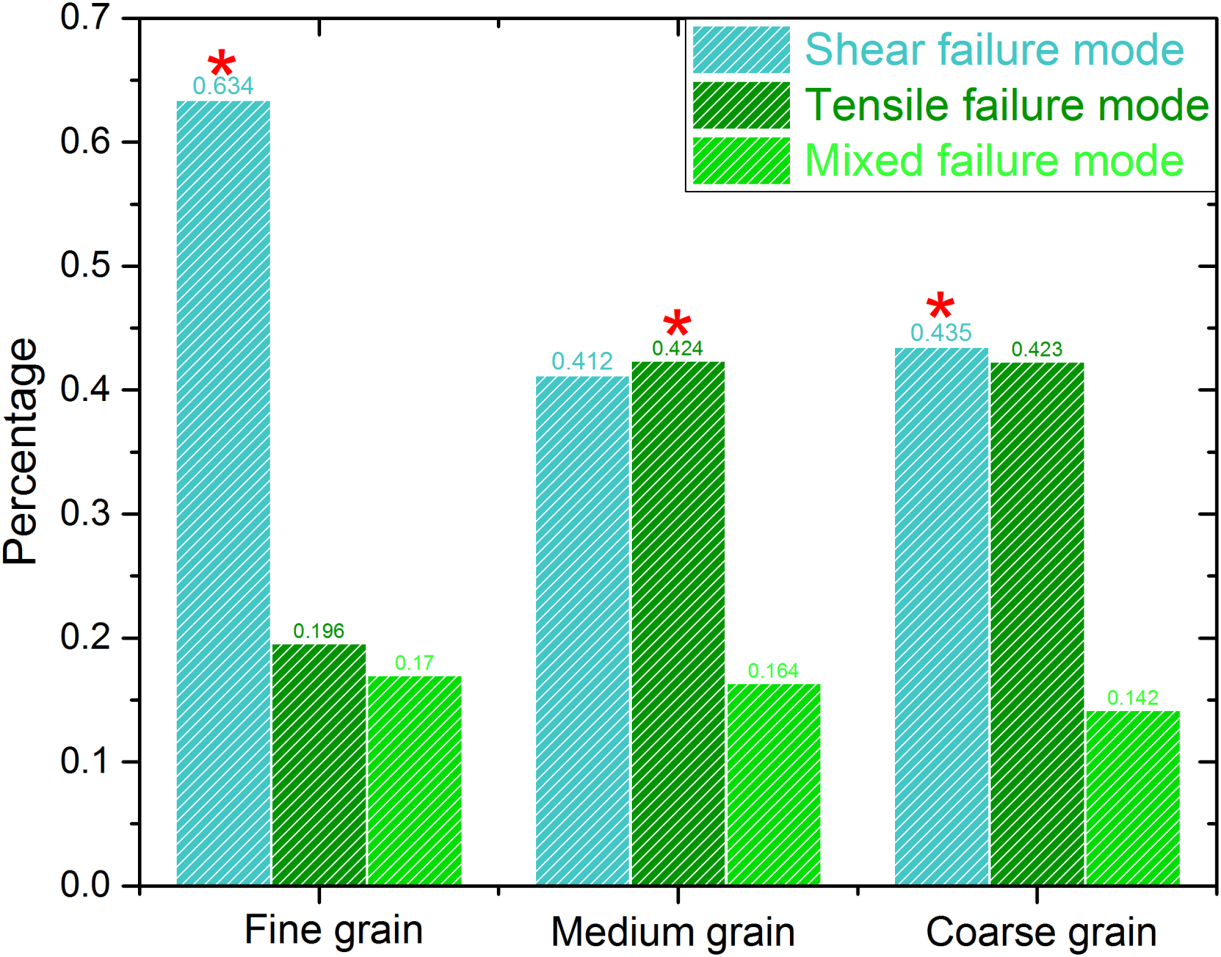
Comparison of 3-p-b fracture types in granite with three kinds of grain sizes.

In the figure, the blue, red and green signals represent the shear, tensile and mixed failure respectively. Fig 6 shows the percent of different failure modes around the notch tip under the 3-p-b test. It can be seen from the figure that the failure mechanism of granite samples is mainly caused by shear failure, and the secondary is tensile failure. To be specific, shear failure accounts for 60% (biggest percentage) under 3-p-b tests of smaller grain size. With the increase of grain size, the tensile failure is generally becoming to a major factor. However, the mixed-failure mode can rarely be found in 3-p-b test.

## Fracture mechanism of notched tip under3-p-b test

### Fictitious crack model for describe the notch tip

**Fig 7:**
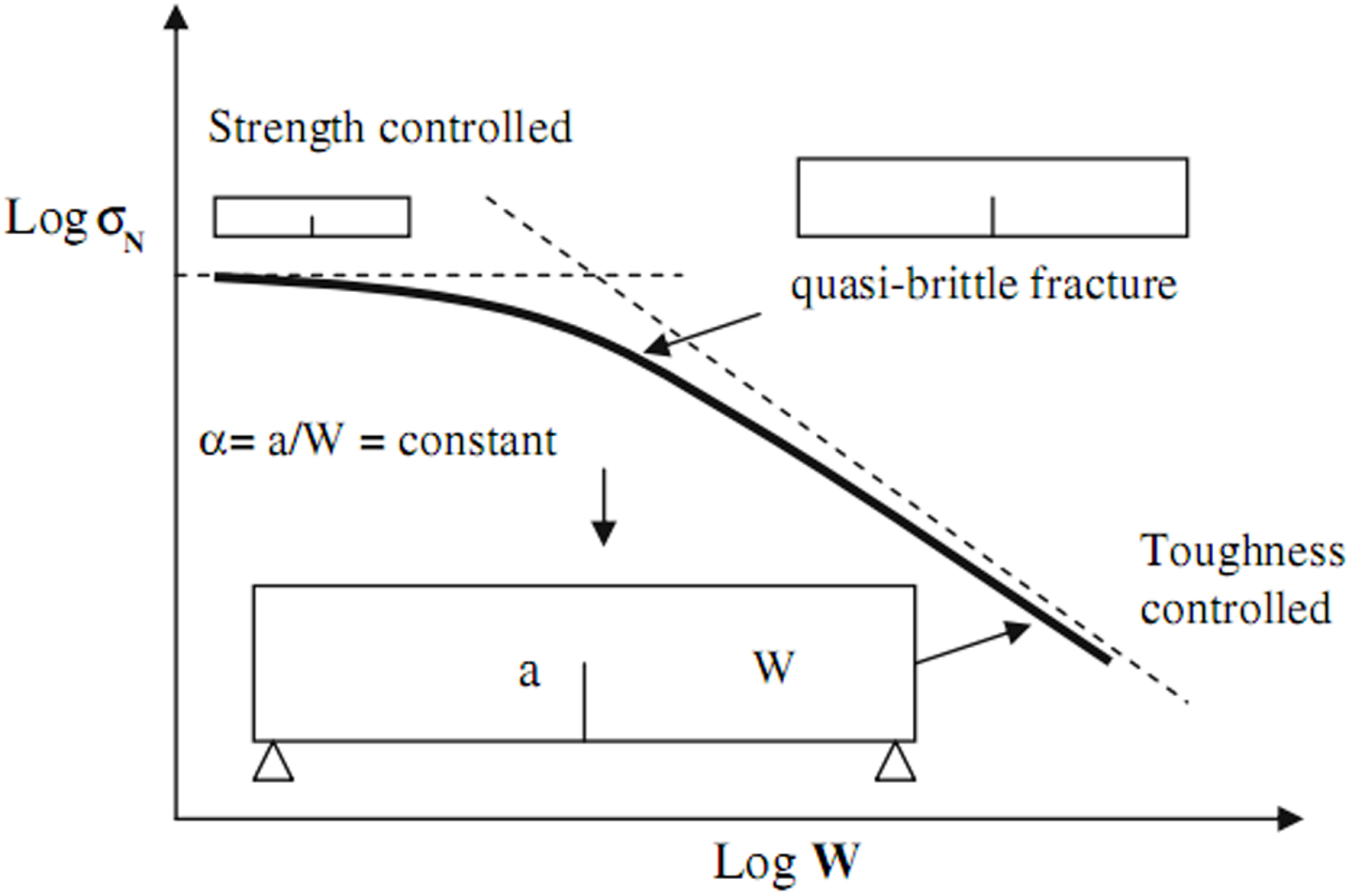
The strength and fracture toughness asymptotic limits under quasi-static load of 3-p-b test.

In this paper, acoustic emission system is used to monitor formation of the micro-cracking initiation and corresponding load displacements of granite. And the fracture process of granite can be divided into five phases under 3-p-b test as Fig 1 illustrated. Also the fracture process zone around notch tip is described by a modified fictitious model [36], where applies cohesive stress to model crack bridge mechanism. The partially and fully developed FPZ are appeared at and after the peak load respectively from the Fig 1. The FPZ measurement is governed by two major failure criteria *f*_*t*_ and *K*_*IC*_, which depends on the notched length. Fig 7 indicates the variation of fracture behavior which is controlled by two asymptotic limits. If *α* is small, or *a*/*W*→0, the fracture process is controlled by tensile strength, while if *α* is large, or *a*/*W*→∞, the fracture process is controlled by fracture toughness.

The fictitious crack model (or “cohesive crack model”) is a general description of deformation properties where fracture process zone (FPZ) is mainly governed by tensile strength. Hoover and Bazant [37] thought that the FPZ can be controlled by the tensile strength and fracture toughness, which depends on the size effect of the specimens. On the other hand, Wang and Hu [36] studied the stress distribution of FPZ with notched specimens under three-point-bending tests and found that the failure controlled ranging from tensile strength to fracture toughness depends on the notched length (“boundary effect”). However the above models cannot take the grain size of the specimens into consideration, this paper link the grain size of the rock samples with fictitious crack growth to describe the FPZ.

### Modified fictitious crack model by bi-linear crack-bridging stress distribution

For the past years, many experts [38, 39] have used the regular crack-bridging stress to describe the partially developed FPZ (or Δ*a*_*fic*_). However, Δ*a*_*fic*_ is relative smaller compared to the crack tip opening displacement around the notch tip when the load ups to peak and it is difficult to measure. This paper introduces the modified fictitious crack model (*a*_*fic*_ = *a*_0_+Δ*a*_*fic*_) to address the question. In order to calculate the modified nominal strength *σ*_*n*_, the bending stress and bending moment can be took into consideration along the crack plane. Linear strain equation is used as Fig 2 shown:

$$\frac{\sigma_{c}/E_{c}}{\sigma_{n}/E_{c}}=\frac{y}{x}$$ (1)

$$y=\frac{\sigma_{c}}{\sigma_{n}}x$$ (2)

In which *x* and *y* represents the distance from the fictitious crack tip and the point of peak load to the middle axis respectively illustrated in Fig 2.

From the figure it can be concluded that:

$$x+y=W-a_{0}-\Delta a_{fic}$$ (3)

The force equilibrium criterion can be written:

$$\frac{1}{2}•\sigma_{c}•y=\frac{1}{2}•\sigma_{n}•x+\Delta a_{fic}•\sigma_{n}$$ (4)

The moment equilibrium criterion is obtained:

$$\begin{array}{ll} \frac{1}{2}P_{\text{max}}•\frac{1}{2}S•\frac{1}{B} & =\frac{1}{2}\sigma_{c}•y•\frac{2}{3}y+\frac{1}{2}\sigma_{n}•x•\frac{2}{3}x+\sigma_{n}•\Delta a_{fic}•(\frac{\Delta a_{fic}}{2}+x)\\ & =\frac{1}{3}\sigma_{c}y^{2}+\frac{1}{3}\sigma_{n}x^{2}+\sigma_{n}•\Delta a_{fic}•(\frac{\Delta a_{fic}}{2}+x) \end{array}$$ (5)

According to Eqs (2)–(5), three unknown parameters (*σ*_*c*,_ *x* and *y*) can be eliminated and the following equation can be deduced:

$$\sigma_{n}=\frac{\frac{S}{B}P_{\text{max}}}{\frac{\left(w-a_{o}-\Delta a_{fic})(w-a_{o}+\Delta a_{fic})+{\left(w-a_{o}-\Delta a_{fic}\right)}^{4}+6\Delta a_{fic}{\left(w-a_{o}-\Delta a_{fic}\right)}^{2}(w-a_{o}\right)}{3{\left(w-a_{o}\right)}^{2}}+2\Delta a_{fic}{}^{2}}$$ (6)

In which *P*_*max*_ can be obtained by 3-p-b test, and nominal strength *σ*_*n*_ can be deduced if Δ*a*_*fic*_ is known. For rock samples, the mineral characterization has an effect on the formation of fictitious crack during the 3-p-b test. This paper linked the value of Δ*a*_*fic*_ with the microstructure of the three kinds of grain sizes, and the bigger average grain size corresponds to the larger FPZ (or Δ*a*_*fic*_). The following equation has been assumed by Wang et al. [36]:

$$\Delta a_{fic}=\lambda •g_{av}$$ (7)

In which paper, *λ* is obtained equal to average grain size of granite, but it is an estimated value and fail to represent the microstructure of three kinds of granites. Therefore the concept of fractal is introduced in this paper to describe the distribution of the minerals on a meso-scale as Eq (8) express:

$$\text{ln}(N/N_{0})=D\text{ln}(r_{\text{max}}/r)$$ (8)

Where *r* represents the grain size of the characteristic mineral; *N* is the number of minerals whose grain size is greater or equal to that of the characteristic mineral *r; N*_*0*_ is the number of the maximum characteristic mineral *r*_*max*_; b is the distribution index of the characteristic mineral, and it also can be regarded as the fractal dimension of the three kinds of granite samples.

**Fig 8:**
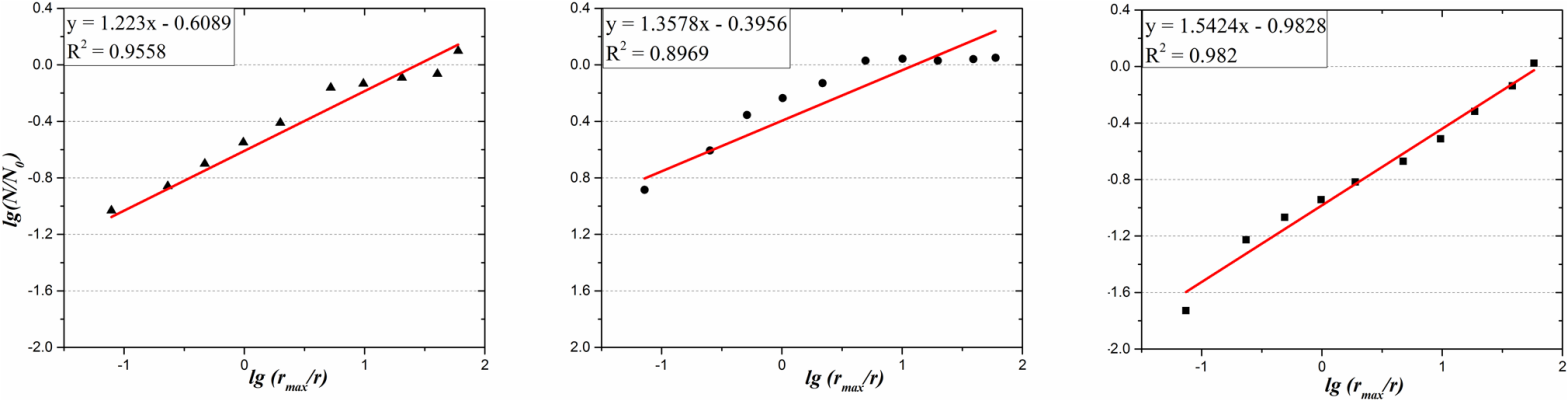
Fractal dimension characterization of granite specimen with three kinds of grain sizes. (a) Grain size = 3mm (b) Grain size = 2.12mm (c) Grain size = 1.01mm.

The grain sizes of three kinds of granite samples can be analyzed by digital image process and *r* values are selected in the range of minimum and maximum values of grain size for each kind of granite respectively. Therefore, the values of fractal dimension can be obtained from the linear fitted curve according to Eq (8) as illustrated in Fig 8. And it is well recognized that the values of three kinds of granite are 1.223, 1.3578 and 1.5425 respectively.

In summary, the fictitious crack growth (Δ*a*_*fic*_) can be calculated by Eq (7) and the results of three kinds of granite with grain size = 1.01, 2.12 and 3mm are correspondence to Δ*a*_*fic*_ = 1.58, 2.87 and 3.66mm.

The peak load *P*_*max*_ is obtained using 3-p-b test and therefore the modified nominal strength *σ*_*n*_ can be calculated by Eq (6) for Δ*a*_*fic*_ = 1.58, 2.87, 3.66mm as illustrated in Table 2.

**Table 2:** Average nominal strength *σ*_*n*_ for different fictitious crack growth Δ*a*_*fic*_.

*a*_0_ / *mm* Notched length	Average nominal strength *σ*_*n*_ (MPa)			Samples tested
	Δ*a*_*fic*_ = 1.58mm	Δ*a*_*fic*_ = 2.87mm	Δ*a*_*fic*_ = 3.66mm	Total (trisection)
0	10.8	10.6	10.4	9
1	10.7	10.5	10.3	9
5	10.4	10.5	10.2	9
10	11.2	11	10.8	9
15	11	10.6	10.4	9
20	10.4	10.1	9.9	9
25	10.1	9.8	9.6	9
30	10.5	9.6	9.4	6
35	10	9.7	9.5	6
40	11.1	10.9	10.7	6
45	11.3	11	11.1	6

### Calculation for tensile strength and fracture toughness with 3-p-b test

The relationship between nominal strength *σ*_*n*_ and tensile strength *f*_*t*_ has been presented in the Hu literature [33, 34], with the derivation of analysis given:

$$\sigma_{n}=\frac{f_{t}}{\sqrt{1+\frac{a_{e}}{a_{\infty }^{*}}}}$$ (9)

Equivalent crack length *a*_*e*_ and specimen geometry *B*(α) can be obtained from Eqs (10) and (11):

$$a_{e}=B(\alpha )a_{0}$$ (10)

$$B(\alpha )={\left(\frac{\text{Y}(\alpha )(1-\alpha {)}^{2}}{1.12}\right)}^{2}$$ (11)

$$\text{Y(}\alpha \text{)}=\frac{1.\text{99}-\alpha \cdot (1-\alpha )(2.15-3.93\cdot \alpha +2.7\alpha^{2}{)}^{3/2}}{\sqrt{\pi }\text{(1}+\text{2}\cdot \alpha )(1-\alpha {)}^{3/2}}$$ (12)

$$a_{\infty }^{*}=0.25\cdot {\left(\frac{K_{IC}}{f_{t}}\right)}^{2}$$ (13)

$$\frac{1}{\sigma^{2}{}_{n}}=\frac{1}{f_{t}{}^{2}}+\frac{1}{f_{t}{}^{2}a_{\infty }^{*}}\cdot a_{e}=\frac{1}{f_{t}{}^{2}}+\frac{4}{K_{IC}{}^{2}}\cdot a_{e}$$ (14)

Where *α* is the ratio of notched length *a*_0_ and the width of samples *w*, *a*_e_ is the equivalent crack length, *a*_∞_* is the characteristic crack length, and *f*_*t*_ and *K*_IC_ represent the tensile strength and fracture toughness of granite samples respectively. The characteristic crack length is a constant with the determination of the tensile strength and fracture toughness, and it can be taken from test standards of fracture toughness based on the American Society of testing and materials [40].

**Fig 9:**
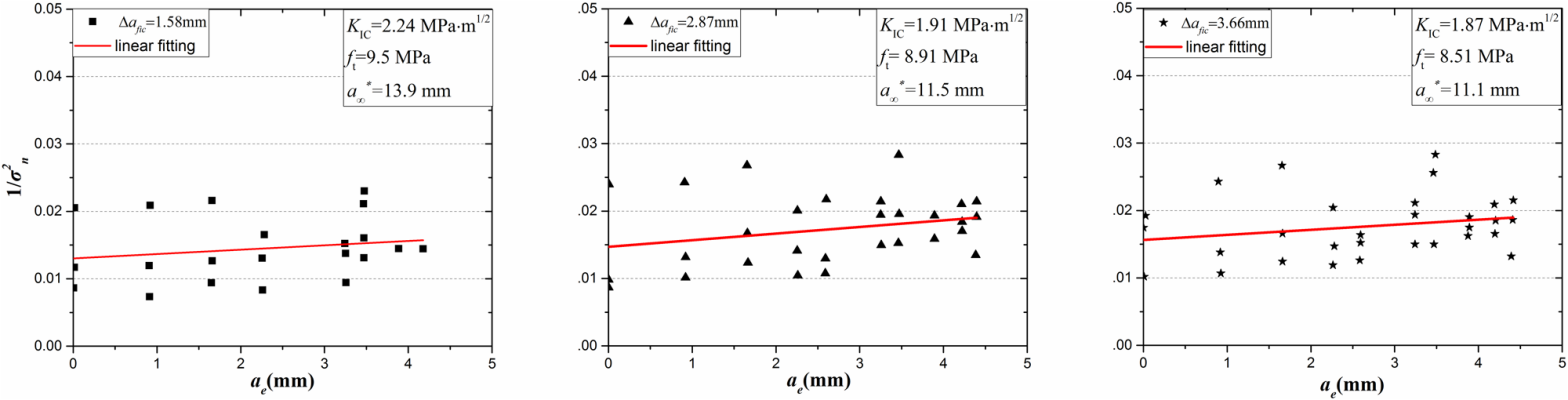
Linear regression results for determination for tensile strength *f*_*t*_ and fracture toughness *K*_IC_.

According to Eqs (9)–(13), it can be deduced that the *f*_*t*_ and *K*_IC_ of granite with different grain sizes can be directly obtained by the linear regression method according to Eq (14) under a simple 3-p-b test during the quasi-static fracture process as Fig 9 shown.

The results of tensile strength and fracture toughness are simulated by linear regression and eliminating the longer notched length (40, 45 mm) which makes it unstable. And also it can be found in Table 3 that the tensile strength and fracture toughness of the three kinds of granite are 9.5MPa and 2.24 MPa∙m^1/2^, 8.91MPa and 1.91 MPa∙m^1/2^ and 8.51MPa and 1.87 MPa∙m^1/2^.

**Table 3:** Tensile strength and fracture toughness for different fictitious lengths at peak load.

Regression conditions		Results	
Δ*a*_*fic*_	Notched length excluded (mm)	*f*_*t*_ (MPa)	*K*_IC_(MPa√m)
1.58	-	9.67	2.23
	45	9.63	2.43
	40 and longer	9.58	2.45
	35 and longer	**9.5**	**2.24**
	30 and longer	**9.5**	**2.24**
	25 and longer	9.61	2.35
2.87	-	9.21	2.12
	45	9.13	2.05
	40 and longer	8.98	1.98
	35 and longer	**8.91**	**1.91**
	30 and longer	**8.91**	**1.91**
	25 and longer	9.15	2.08
3.66	-	8.67	2.11
	45	8.64	2
	40 and longer	8.42	1.91
	35 and longer	**8.51**	**1.87**
	30 and longer	**8.51**	**1.87**
	25 and longer	8.54	1.69

## Numerical simulation experiment

### Brief description of RFPA-DIP software

A numerical software RFPA-Dip (Rock Failure Process Analysis-Digital Image Process) version is developed to describe the microstructure of the rock sample based on the RFPA-2D version. This study uses this code to simulate the fracture process of rock samples for three reasons:(1) the microstructure of the rock can be characterized and minerals inside the specimen can be distinguished from each other using threshold values based on the digital image process, (2) The relationship between the fictitious crack model and the grain size can be built by using RFPA-Dip version to describe the stress distribution at the notch tip under three-point-bending test, (3) RFPA-Dip code is capable of simulating the fracture process of the rock specimens on the micro-scale and explaining the failure mechanism combined with AE monitoring during the fracture process.

### Heterogeneity characterization of granites and model validity

**Fig 10:**
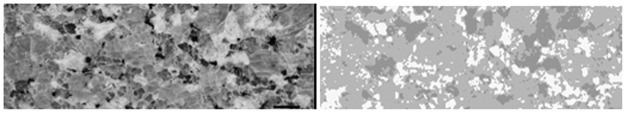
(a) Digital image of coarse grain and (b) Simulation model based on digital image of specimen.

This paper uses the numerical simulation RFPA-Dip version, developed by CRISR team in the Northeastern University, to simulate the fracture process of three kinds of granites under 3-p-b tests. The rock heterogeneity can be characterized by digital image processing (DIP) technology, which is also used to classify the mineral grains on a micro-scale. Fig 10 illustrates the surface image of the selected granite specimen in this paper, whose pixel size is 280p×70p in equal proportion to the model size (280mm×70mm). Image processing conducts multi-threshold segmentation in HIS (Hue, Saturation, and Intensity) color space with variation of the brightness I value, and different thresholds are set to distinguish the mineral grains.

**Fig 11:**
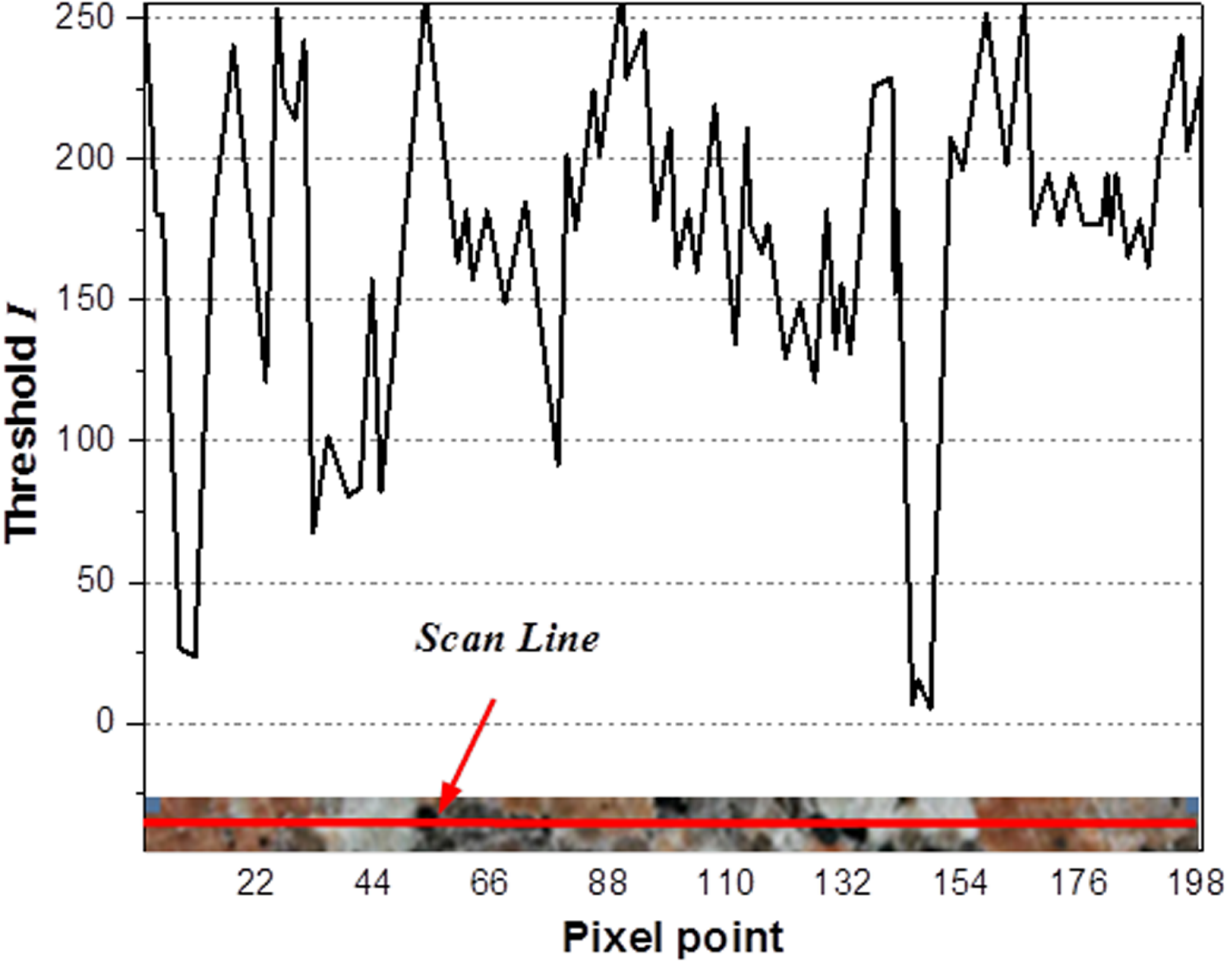
Threshold value (*I*) change in the scanning line.

The granite samples shown in the figure are carried out with threshold selection. Different color regions stand for different kinds of mineral particles, which mean that the red, black and white regions represent feldspar, mica and quartz grains respectively. Fig 11 shows the variation of I value of the mineral medium on a cross section of the numerical images. After repeated experiments, the range of threshold values is set to 50 and 200, and then the threshold I is divided into three sections (0~50, 50~200, 200~255) to distinguish different minerals.

Fig 10(b) shows the digital image of the granite samples processed by threshold segmentation, which can be used to describe the heterogeneity on a micro-scale. The digital image is composed of a rectangular array of pixels in nature, whose principle is to assign the values of mineral grains in accordance with variable colors of each pixel. Therefore the method is an effective way to characterize the heterogeneity of the rock samples.

**Fig 12:**
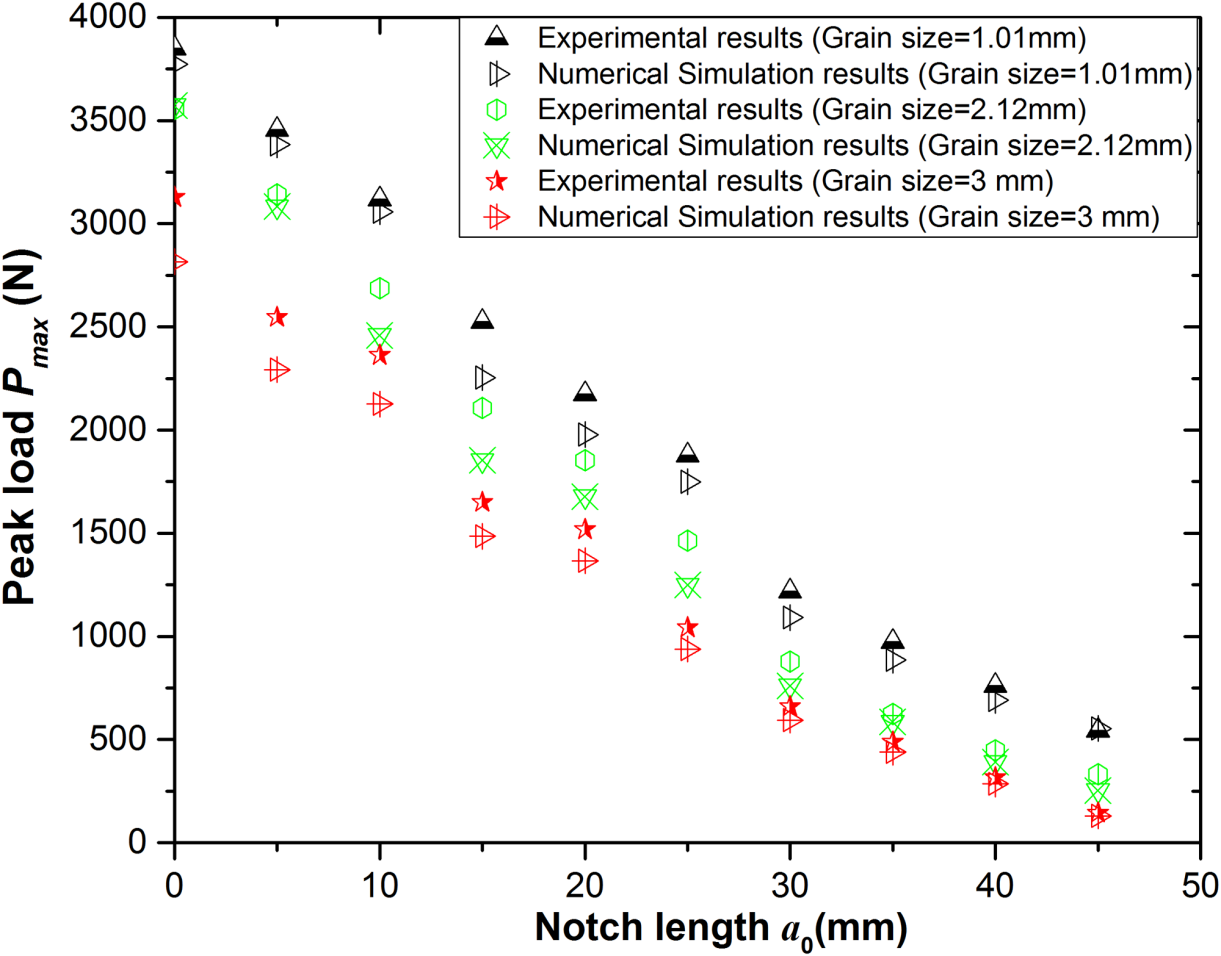
The results contrast between numerical simulation and physical experiments under 3-p-b tests.

This study selects the granite specimen with three kinds of grain size for an example to validate the numerical model by comparing the experimental results. The numerical model is simplified as the plane stress problem, and also load control is adopted with an increment of 0.001 mm/step. The comparison results of peak loads under 3-p-b tests using the methods of numerical simulation and physical experiments are shown in Fig 12.

### Numerical results of 3-p-b test

**Fig 13:**
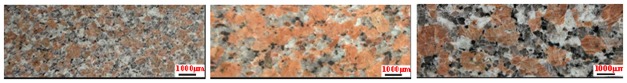
Granites with three kinds of grain sizes processed by digital image. (a) grain size = 1.01mm (b) grain size = 2.12mm (c) grain size = 3mm.

**Fig 14:**
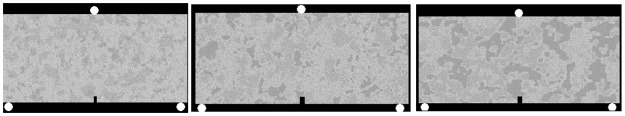
The 3-p-b numerical model of three kinds of granite with notched length.

Granite samples selected in the paper are transformed into digital picture and conducted 3-p-b tests using RFPA-Dip version as Fig 13 illustrated. Moreover a numerical model is constructed based on the digital image of three kinds of granites (length × width = 280×70mm), whose notched length (1,5,10…40mm) is prefabricated in Fig 14.

**Fig 15:**
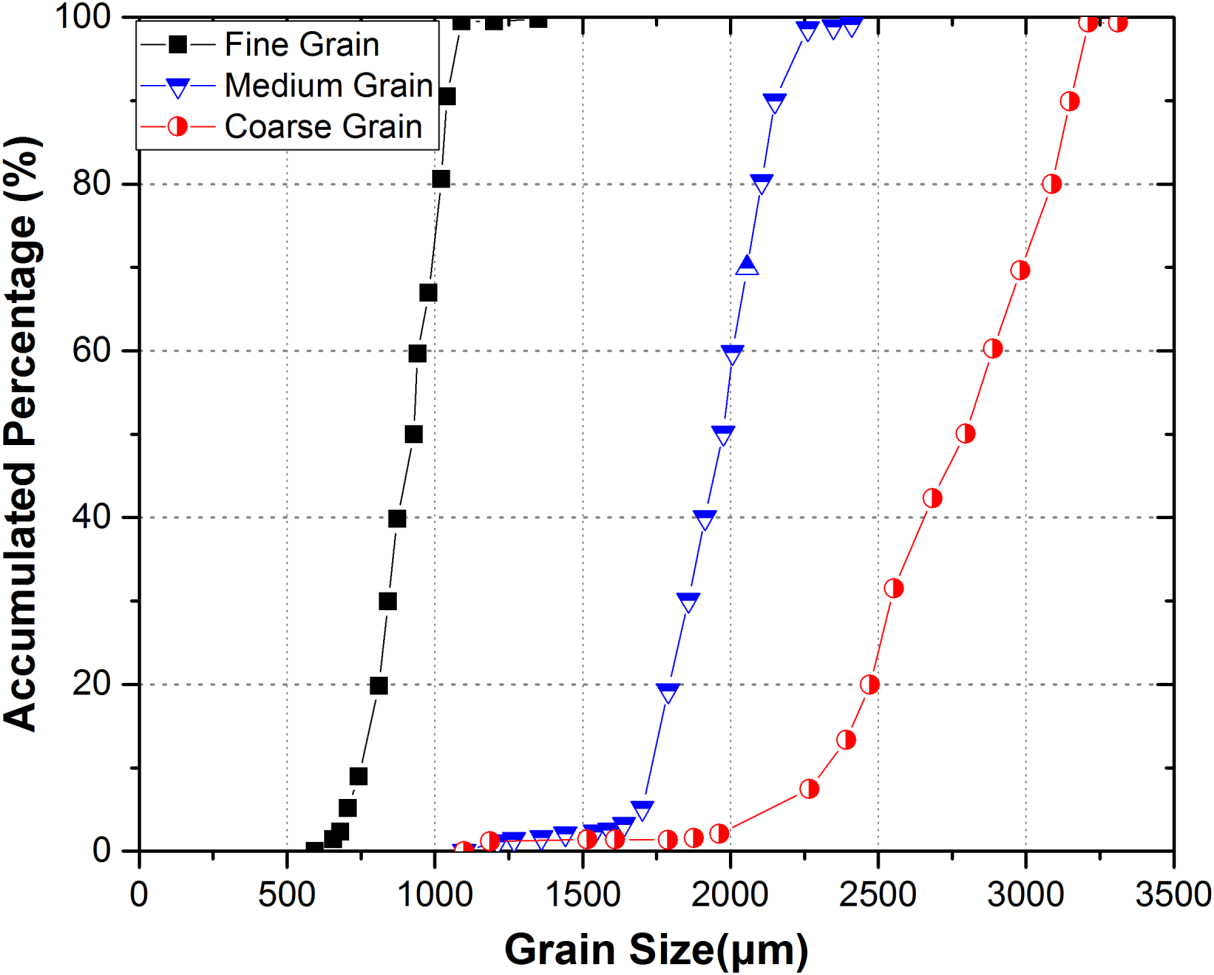
The grain size distribution of three kinds of granite samples.

Fig 15 shows statistics results of grain size distribution on a micro-scale and it can be calculated that the average grain sizes of three kinds of granite are 1.01, 2.12 and 3 mm respectively.

Table 4 shows the basic mechanical parameters of minerals inside the rock specimens. It is noticeable that this paper regards a single mineral particle as a homogeneous medium, irrespective of the heterogeneity.

**Table 4:** Micro-mechanical parameters in RFPA [41].

Mineral types	Elastic modulus (GPa)	Tensile strength (MPa)	Possion’s ratio	Compressive-tensile strength ratio
Quartz	96	373	0.08	15
Feldspar	67	172	0.27	12
mica	40	90	0.25	10

**Fig 16:**
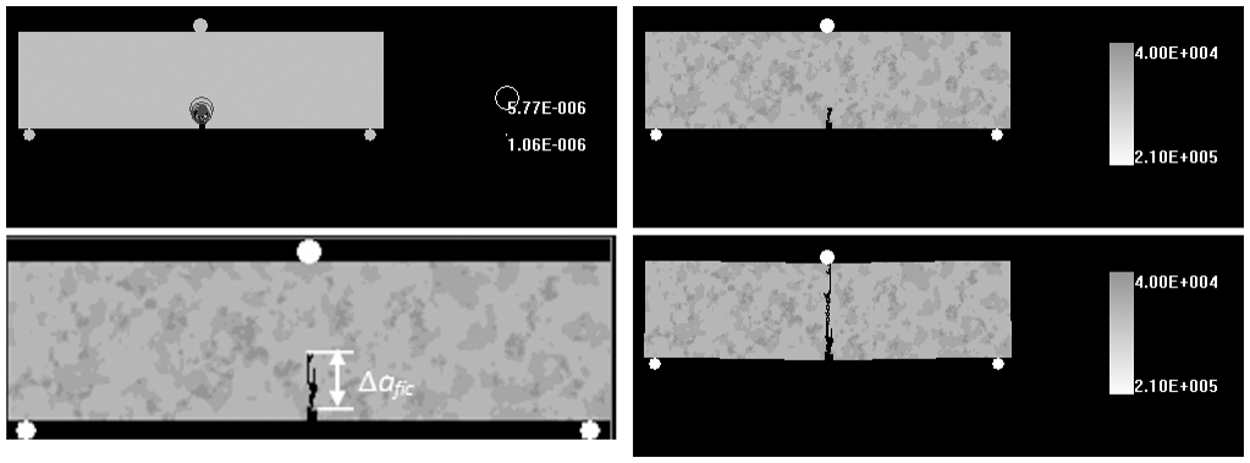
Failure process of 3-p-b test (notch length = 1mm) of granite disc with numerical simulation. (a) 200^th^ load step (b) 348^th^ load step (c) 380^th^ load step (d) 500^th^ load step.

**Fig 17:**
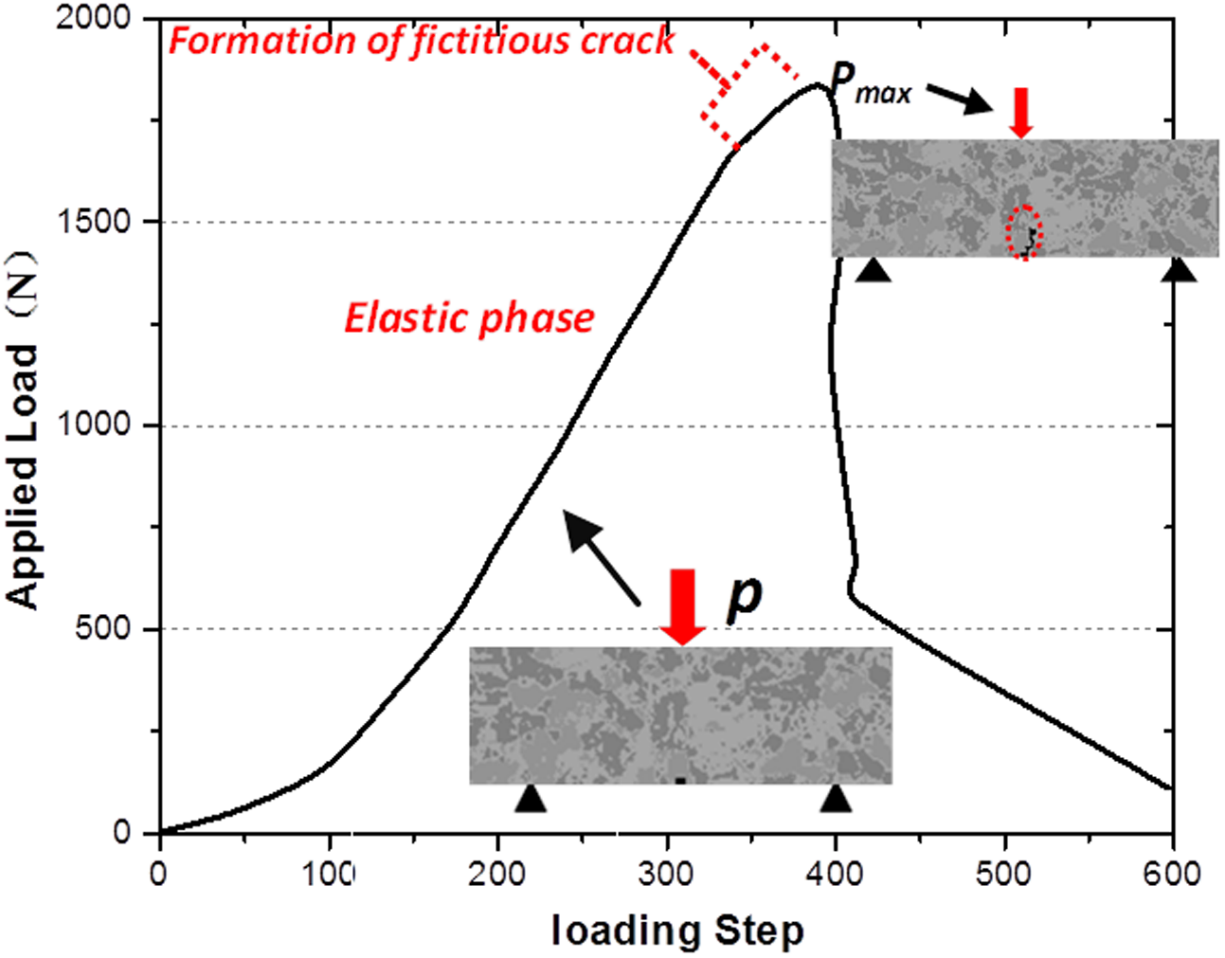
The relation curve of applied load and loading step under 3-p-b numerical test.

The 3-p-b numerical models are performed to explain the failure mechanism of the granite under quasi-static loads and the following discussions are illustrated in a case of the notched length = 1 mm. As Fig 16(a) shown, the accumulated damage in the notch tip is caused by tension stress (red circle) as the displacement loading ups to the 300th steps, which conforms to the characteristics of fictitious crack model proposed by Hillerborg. Moreover, this phase also corresponds to the elastic loading region in Fig 17. The microcracks formed by damage accumulation initiate and growth gradually around the notch tips when the displacement loading ups to 348th step as Fig 16(b) shown. It also can be seen from Fig 16 that this phase corresponds to the plastic loading stage and the formation of the fictitious crack. The 3-p-b numerical test reaches peak load as the loading is up to 380th step and at this moment the crack growth length just exceeds the fracture process zone (Δ*a*_*fic*_) as Fig 16(c) illustrated. With the continuation of the quasi-static loading, the macro-cracks show instable propagation and eventually lead to the failure of the granite samples as shown in Fig 16(d).

Fig 17 shows that the fracture period from microcracks initiation to instable growth and coalescence is relative short (348th~380th step), which can represent the brittle behavior of granite samples. Moreover, it can be observed that the number of cracks is small, branch cracks can rarely been found and fracture surface fails to the zigzag modes.

**Fig 18:**
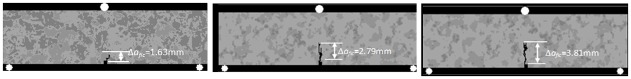
The values of fictitious crack growth Δ*a*_*fic*_ at the peak load under 3-p-b test. (a) Fine grain size = 1.01mm (b) Medium grain size = 2.12mm (c) Coarse grain size = 3mm.

Fig 18 shows the length of fictitious crack growth (Δ*a*_*fic*_) of three kinds of granite specimens at the peak load under 3-p-b test, and it can be found that the results of the numerical simulation are similar to those of the model prediction. All the 3-p-b specimens have an obvious fictitious crack growth with a length of 1~4 mm.

### Comparison of numerical tests and model prediction results

**Fig 19:**
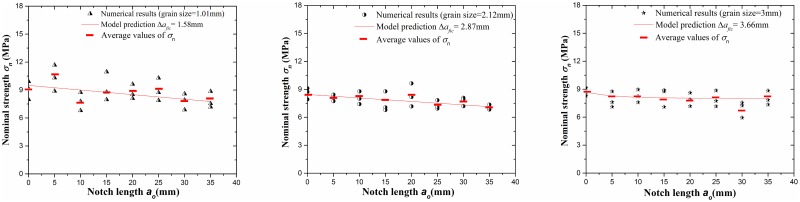
The contrast of the nominal strength *σ*_*n*_ between numerical tests and model prediction.

The numerical tests of 3-p-b samples (length × width = 200mm×50mm) with three kinds of selected granites are conducted by using RFPA-Dip version. Because the tensile strength and fracture toughness have been calculated from the physical tests in above sections, the peak loads and nominal strengths can be predicted by using Eqs (6), (9) and (14). Therefore, the change of nominal strength *σ*_*n*_ with the notch length *a*_0_ is shown in Fig 19 and also it can represent a comparison of the numerical tests and model prediction under 3-p-b test to determine the *σ*_*n*_. The results show that the agreement between two methods is improved with the increase of grain size or fictitious crack growth Δ*a*_*fic*_.

**Fig 20:**
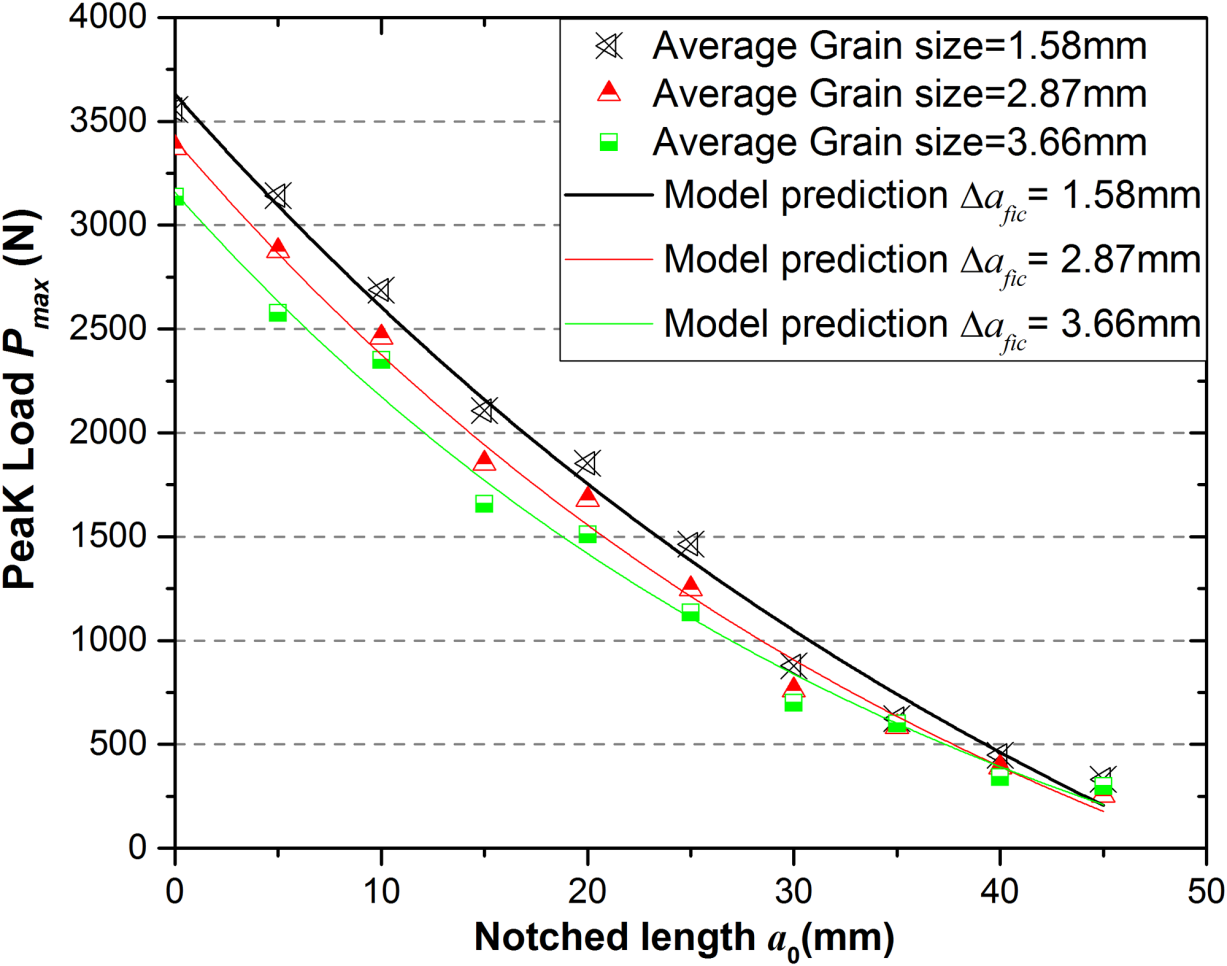
Contrast of the peak load between model prediction and numerical experiments under 3-p-b test.

The peak loads *P*_*max*_ for variations of notch length *a*_0_ obtained by numerical tests and model prediction are represented in Fig 20 with three kinds of granites. The agreement of the two methods is more suitable when the fictitious crack growth is longer.

## Conclusion

In summary, this paper presents a new method to calculate the tensile strength and fracture toughness of three kinds of granite samples with different notch lengths by using three-point-bending (3-p-b) tests. A modified fictitious model is introduced to describe the fracture process zone (FPZ) around the notch tip and it can link the FPZ with grain size on a micro-scale at the peak load during quasi-static fracture process. Moreover, acoustic emission system can be used to monitor the failure mechanism of samples around the notch tip. The combination methods of physical and numerical tests are performed to validate the model and also acoustic emission system is introduced to monitor the fracture mechanism around the notch tip. The numerical simulation RFPA-Dip version is developed to characterize the microstructure of the rock specimens and can be used to conduct the 3-p-b tests, in which process only peak loads and average grain size of three kinds of samples are needed. The results can be concluded that: (1) a modified fictitious crack model is presented and used to link the microstructure of the rock with fracture process around the notch tip and it is an effective method to calculate the tensile strength and fracture toughness at the peak loads of 3-p-b tests under quasi-static loadings; (2) fracture mechanism is governed by grain size of the rock samples and moreover it is shown that the smaller grain size corresponds to the shear failure and the percentage of tensile failure is gradually larger with the increase of the grain size. (3) fractal concept can be introduced to describe the microstructure and heterogeneity of granites and calculate the fictitious crack growth; (4) numerical experiments of above 3-p-b samples are conducted to understand the fracture mechanism of three kinds of granites and validate the theoretical model in contrast to the results of physical tests and model prediction.

## Supporting information

S1 File Data of Fig 3(b). (XLSX)

S2 File Data of Fig 6. (XLSX)

S3 File Data of Fig 9. (XLSX)

S4 File Data of Fig 11. (XLSX)

S5 File Data of Fig 12. (XLSX)

S6 File Data of Fig 15. (XLSX)

S7 File Data of Fig 19. (XLSX)

S8 File Data of Fig 20. (XLSX)
